# Physical and mental health outcomes including behavior and attitudes in people having social contacts with COVID-19 patients

**DOI:** 10.1371/journal.pone.0245945

**Published:** 2021-02-02

**Authors:** Zijun Xu, Dexing Zhang, Dong Xu, Xue Li, Yao Jie Xie, Wen Sun, Eric Kam-Pui Lee, Benjamin Hon-Kei Yip, Phoenix Kit-Han Mo, Shuiyuan Xiao, Samuel Yeung-Shan Wong

**Affiliations:** 1 JC School of Public Health and Primary Care, The Chinese University of Hong Kong, Hong Kong, China; 2 Global Health Institute, School of Public Health and Institute of State Governance, Sun Yat-sen University, Guangzhou, China; 3 Department of Pharmacology and Pharmacy, Li Ka Shing Faculty of Medicine, The University of Hong Kong, Hong Kong, China; 4 Department of Medicine, Li Ka Shing Faculty of Medicine, The University of Hong Kong, Hong Kong, China; 5 School of Nursing, The Hong Kong Polytechnic University, Hong Kong, China; 6 Xiangya School of Public Health, Central South University, Changsha, China; Nagoya University Asian Satellite Campuses Institute, Nagoya University Graduate School of Medicine, MYANMAR

## Abstract

The novel coronavirus (COVID-19) infection has spread worldwide since late 2019. People who have social contacts with COVID-19 patients might be at higher risk of physical or mental health problems. This study investigated whether people who had social contacts with COVID-19 patients would have poorer physical or mental outcomes, and different attitudes and behaviors. Chinese adults were recruited to fill in an online survey using snowball sampling during 21^st^-26^nd^ February 2020. Physical symptoms, psychological outcomes, quality of life, COVID-19 related attitudes, and behaviors were measured. The differences in the outcomes between participants who had COVID-19 social contacts and those who had not were analyzed. The survey included 1,447 non-infected eligible participants. Among those, 173 (12.0%) reported at least one confirmed/suspected case in their social contacts. In the multiple regression adjusted for demographic data, the presence of confirmed/suspected infection cases in one’s social contacts was significantly associated with poorer physical and mental outcomes, lower health-related quality of life, and different COVID-19 related attitudes and behaviors (*p*<0.05). In conclusion, people who had social contacts with COVID-19 patients were at risk of adverse health outcomes. Future studies are needed to understand the long-term impacts. Similarly, strategies to improve health outcomes for these people are needed.

## Introduction

The novel coronavirus (COVID-19) infection has spread worldwide since first detected in Wuhan, China in December 2019 [1]. As of September 2020, more than 33 million people were infected with COVID-19 worldwide, causing over one million deaths [2]. In addition to physical health complications and deaths, COVID-19 and its related quarantine measures have caused widespread mental health deteriorations in many populations [3]. Therefore, COVID-19 has overburdened the healthcare systems, and the scarce medical resources should be diverted to screen and treat the higher risk populations [4].

Previous studies had shown that healthcare workers and people with pre-existing chronic diseases were at higher risk of health problems when exposed to COVID-19 [5–9]. We hypothesize that people who have social contacts with COVID-19 patients are also at higher risk of having physical/mental health problems. This is first because people who had social contacts with COVID-19 patients may worry about being infected by the highly infectious virus; at the same time, they may worry about the health of their infected relatives or friends. Around 40% of people having close contact with infected friends and family members caught the infection [10]. Furthermore, people who had social contacts with COVID-19 patients might be stigmatized as infectious [11]. Finally, these people may be strictly quarantined according to the government’s legislation, which may lead to loneliness, boredom, and a change in health-related behaviors (e.g., reduction in exercise [12]). Sun et al. showed that college students who had infected relatives or acquaintances were more likely to suffer from anxiety, and people who traveled to Wuhan or had contacts with people from Wuhan were more likely to suffer from post-traumatic stress disorder (PTSD) [13, 14]. To date, there is a lack of studies that comprehensively investigate whether people who have social contacts with COVID-19 patients have poorer physical and mental health, diminished quality of life, and different COVID-19 related attitudes and behaviors.

This study aimed to fill the gap and determine whether people who had social contacts with COVID-19 patients had poorer physical and mental health and lower quality of life. We hypothesized that people who had social contacts with COVID-19 patients are more prone to develop psychological and physical symptoms. This knowledge will help determine the allocation of scarce medical resources to these high-risk individuals. Essential factors to adapt to the COVID-19 epidemic, such as lifestyles (e.g., exercise levels), social support, and self-efficacy, were also measured [12]. This study was conducted about 1.5 months after the COVID-19 outbreak in Wuhan City when 76,288 patients were infected by the virus [15].

## Materials and methods

This study was approved by the Survey and Behavioural Research Ethics Committee of the Chinese University of Hong Kong. The study was also registered in the World Health Organization recognized registry (ChiCTR2000030223).

### Participants

Participants were recruited to fill in the online survey between February 21–26, 2020. Participants were eligible if they were Chinese and aged over 18 years. Participants were excluded if they were infected by COVID-19. Potential participants were recruited using snowball sampling via WeChat, one of the most popular mobile applications (Apps) for communications in China. The participants were encouraged to pass on the survey link (accessible on the Wenjuanxing website (www.wjx.cn)) and posters to their friends and family members. Participants voluntarily filled in the survey after reading the informed consent shown at the beginning of the survey.

To enhance the response rate, respondents were entered into a lucky draw of 1–10 RMB after completing the survey. To avoid duplicated responses, one device could only respond to the survey once. Upon completion, the results of physical and mental distress levels were provided back to the participants. A list of available health community resources was also provided to those with possible physical or mental distress.

### Survey instrument

The questionnaire was designed by a group of academics in clinical medicine, psychiatry, nursing, epidemiology, and health service management. The survey's feasibility, clarity, and acceptability were confirmed by piloting with 20 Chinese adults with different demographics backgrounds (e.g., age and educational level). Minor amendments were made according to the comments received. The questionnaire can be found under, S1 File.

#### Demographics

Social contacts with suspected or confirmed COVID-19 patients (e.g., in friends, family, colleagues, and close neighbors) was self-reported. Other demographic data, including age, sex, marital status, work, educational level, self-reported household income level according to the local economy, and residential location, were also collected.

#### Outcomes

Depressive, anxiety and post-traumatic stress disorder (PTSD) symptoms were assessed using the validated Chinese versions of the two-item Patient Health Questionnaire (PHQ-2), the two-item Generalized Anxiety Disorder Questionnaire (GAD-2), and two of the items from the Clinician-Administered PTSD Scale (recurrent dreams and avoidance about the COVID-19 epidemic), respectively [16–18]; Scores of ≥3 suggested possible presence of depression/anxiety disorders/PTSD [19, 20]. The presence of suicidal ideation was detected by question number nine in the 9-item Patient Health Questionnaire (PHQ-9) [17]; Scores from 1 (several days) to 3 (nearly every day) indicated the presence of suicidal ideation. Meaning in life was measured by one item in the validated Chinese Purpose in Life test (CPIL), which asked about the importance of personal existence on a 7-point scale from 1 (utterly meaningless and without purpose) to 7 (very purposeful and meaningful) [21]. Somatic symptoms were measured by the validated Chinese 15-item Patient Health Questionnaire (PHQ-15) [22]; A higher score indicated more somatic symptoms (range from 0–30).

Physical health was assessed by the number of COVID-19 related symptoms (e.g., fever, cough, sore throat) in the previous four weeks. Furthermore, the participants rated their overall health ranging from 1 (poor) to 5 (excellent). Similarly, life satisfaction was rated on a 7-point Likert scale ranging from 1 (very dissatisfied) to 7 (very satisfied) in response to the question “Are you satisfied with your life?”. Health-related quality of life (HRQOL) was measured by the validated Chinese version of the European quality of life 5-dimension (EQ5D-5L) Questionnaire, which also included a visual analog scale (EQ-VAS). Higher scores signified higher HRQOL [23].

As participants who had social contacts with COVID-19 patients may have different perceptions of the COVID-19 epidemic and different daily lives, the questionnaire also asked about (i) their perceptions such as their perceived risk of being infected and perceived time needed to control the epidemic; (ii) impact on their daily lives such as expenditure for personal infection prevention (e.g., buying disinfectants) and health-seeking behavior (e.g., visiting a doctor in prior four weeks); and (iii) their loneliness level, which was measured by the three-item Chinese validated UCLA Loneliness Scale (UCLA-3) (score range 3–9); higher scores represent higher levels of loneliness [24].

Covariates to health outcomes such as hours of moderate/vigorous exercise, hours of sedentary time, diet, screen time, social support, and self-efficacy were also asked. Their diet was measured by the number of days of having different kinds of food in the past week [25]. Self-efficacy was rated on a 4-point scale from 1 (not at all true) to 4 (exactly true), in response to the statement “I can usually handle whatever comes my way”, which was extracted from the General Self-Efficacy Scale [26]. Social support was measured by one item in the Edmonton Frail Scale “When you need help, can you count on someone to fulfill your needs?”; the possible answers included “always”, “sometimes”, and “never” [27].

Quality control was conducted by reliability and logic check as well as a minimal responding time. To check for reliability of answers, the survey had set two repeated questions at the beginning and the end of the survey in a different order. Participants were expected to provide the same or similar answers (score difference no more than one point). Only those surveys that passed the logic checks (e.g., those who answered no drinking in the past year should not answer their drinking frequency as once or more in the consequent question) and at least ≥250 seconds was spent to finish the entire survey, were considered valid.

### Statistical analysis

The baseline characteristics were presented using frequency (and percentage) together with their 95% confidence intervals (CIs) and mean (and standard deviation (SD) for categorical and continuous variables, respectively. Chi-square test (*χ2*) and two-sample t-test were used to compare the differences between those participants who had social contacts with COVID-19 patients and those who had not for categorical data and continuous data, respectively. Statistically significant outcomes, as defined by *p*<0.05, were included in the linear regression model or ordinal logistic regression model. The models were adjusted for age, gender, marital status, education, income, and employment. The strength of associations between COVID-19 patients' presence in one’s social circle and variables of interest were estimated by the adjusted odds ratio (OR) or adjusted coefficient together with their corresponding 95% CIs. As the program did not allow any empty answers, there was no missing data. Sample size calculation was based on the method that at least 10 participants to be included for one independent variable with interests in regression models. All data were analyzed using Stata version 13.1 (StataCorp. 2013. Stata Statistical Software: Release 13. College Station, TX: StataCorp LP.).

## Results

A total of 1742 responses were received, among those, 295 responses were excluded. Two hundred and sixty-eight were excluded as their responses did not meet the quality control standard; Eighteen participants were excluded because of being underage; and nine participants were excluded because of COVID-19 infection. Therefore, 1447 participants were included in the analysis, with an eligibility rate of 83.1%.

The mean age of the participants was 33.9±10.5 years old. The majority of participants were female (59.3%), married (59.5%), employed (69.5%), and obtained a bachelor’s degree or above (72.5%). However, only 110 participants (7.6%) lived in Hubei province. Among the eligible participants, 12% had social contacts with COVID-19 patients (i.e., confirmed or suspected infection cases among their family members, acquaintances, and neighbors) (Table 1).

**Table 1:** Social demographic characteristics of the participants in the study (n = 1447).

Characteristics	Mean±SD or No. (%)
Age (years)	33.9±10.5
Gender (Female)	858 (59.3)
Marriage (Married)	861 (59.5)
Job (Employed)	1006 (69.5)
Education (Bachelor degree or above)	1049 (72.5)
Income level (Average to high)	1125 (77.7)
Current residence (City)	1144 (79.1)
Past-year residence (City)	1279 (88.4)
Current location (Hubei province)	110 (7.6)
Cases within social contacts	173 (12.0)

In the univariate analysis, participants who had social contacts with COVID-19 patients were more likely to have more depressive symptoms, anxiety symptoms, suicidal ideation/thoughts, PTSD symptoms, somatic symptoms, poorer meaning in life, loneliness, lower HRQOL, more COVID-19 related symptoms, and lower life satisfaction (Table 2). After controlled for demographic data and covariates in the multiple regression analysis, these factors, except for anxiety scores, were still statistically significantly associated with having social contacts with COVID-19 patients (Table 3).

**Table 2:** Univariate analysis on quality of life, various health profiles, COVID-19 related factors and their association with suspected or confirmed infection cases within one's social contacts.

Characteristics	Mean±SD or No. (%, 95% CI)			*p*
	Total (n = 1447)	No case within social contacts (n = 1274)	Cases within social contacts (n = 173)	
**Mental and physical health**				
Positive PHQ-2 (≥3)	161 (11.1, 9.6–12.9)	128 (10.0, 8.5–11.8)	33 (19.1, 13.9–25.6)	<0.001***
Positive GAD-2 (≥3)	108 (7.5, 6.2–8.9)	88 (6.9, 5.6–8.4)	20 (11.6, 7.6–17.2)	0.029*
Suicidal or self-harm thoughts	114 (7.9, 6.6–9.4)	84 (6.6, 5.4–8.1)	30 (17.3, 15.2–26.9)	<0.001***
Mild to extreme PTSD symptoms (3–10)	487 (33.7, 31.3–36.1)	410 (32.2, 29.7–34.8)	77 (44.5, 37.3–52.0)	<0.001***
PHQ-15	4.0±4.0	3.7±3.8	5.5±5.3	<0.001***
Meaning in life	5.8±1.3	5.8±1.3	5.6±1.5	0.001**
UCLA-3	3.8±1.3	3.8±1.2	4.2±1.5	0.002**
Past-4-week COVID-19 related symptoms (≥1)	448 (31.0, 28.6–33.4)	368 (28.9, 26.5–31.4)	80 (46.2, 39.0–53.7)	<0.001***
**Quality of life and life satisfaction**				
EQ5D	0.91±0.14	0.92±0.12	0.83±0.25	<0.001***
EQ-VAS	82.8±18.8	83.5±17.7	77.2±24.5	0.001**
Life satisfaction	4.7±1.6	4.7±1.6	4.4±1.6	0.033*
**COVID-19 related factors**				
Overall self-reported negative impact	992 (68.6, 66.1–70.9)	861 (67.6, 64.9–70.0)	131 (75.7, 68.8–81.5)	0.030*
Worry about infection	1139 (78.7, 76.5–80.7)	990 (77.7, 75.3–79.9)	151 (87.3, 81.5–91.5)	0.011*
Perceived high risk for infection	217 (15.0, 13.2–16.9)	163 (12.8, 11.1–14.7)	54 (31.2, 24.8–38.5)	<0.001***
Perceived long control time(>6 months)	171 (11.8, 10.3–13.6)	142 (11.1, 9.5–13.0)	29 (16.8, 11.9–23.0)	0.032*
More expenditures for infection prevention (e.g. buying PPEs, disinfectants, medicines) (≥200 RMB)	603 (41.7, 39.3–44.4)	514 (40.4, 37.7–43.0)	89 (51.5, 44.0–58.8)	0.005**
Past-4-week visiting a doctor	144 (10.0, 9.0–12.1)	105 (8.2, 6.9–10.0)	39 (25.4, 17.0–29.3)	<0.001***
Delayed treatment due to COVID-19	103 (7.1, 5.9–8.6)	85 (6.7, 5.4–8.2)	18 (10.4, 6.7–15.8)	0.073
High perceived self-efficacy (No matter what happens to you, you can handle it easily. Moderately to exactly true)	1041 (71.9, 69.6–74.2)	930 (73.0, 70.5–75.4)	111 (64.2, 56.8–70.9)	0.015*

EQ5D: European quality of life 5-dimension (EQ5D) Questionnaire; EQ-VAS: European quality of life visual analog scale; GAD-2: anxiety symptoms, two-item Generalized Anxiety Disorder Questionnaire; PHQ-2: depressive symptoms, two-item Patient Health Questionnaire; PHQ-15: somatic symptoms, 15-item Patient Health Questionnaire; PPE: personal protective equipment; PTSD: post-traumatic stress disorder; UCLA-3: three-item UCLA Loneliness Scale (range: 3–9). Chi-square test (χ2) and two-sample t-test were used for categorical data and continuous data, respectively.

*P<0.05

**P<0.01

***P<0.001

**Table 3:** The effect of suspected or confirmed infection cases in one’s social circles on quality of life and other variables of interest in multiple regression.

Outcomes	Adjusted*β* coefficient / OR	95% CI	*p*
**Binary variables**^**b**^	**Adjusted OR**	**95% CI**	***p***
Positive PHQ-2	2.062	(1.340, 3.175)	0.001**
Positive GAD-2	1.557	(0.917, 2.641)	0.101
Suicidal or self-harm thoughts	2.708	(1.686, 4.348)	<0.001***
Positive PTSD	1.665	(1.200, 2.310)	0.002**
Past-4-week COVID-19 related symptoms (≥1)	2.075	(1.492, 2.888)	<0.001***
Overall self-reported negative impact	1.388	(0.952, 2.023)	0.089
Worry about infection	1.844	(1.167, 2.914)	0.009**
Perceived high risk for infection	3.055	(2.103, 4.440)	<0.001***
Perceived long control time (>6 months)	1.488	(0.952, 2.325)	0.081
High expenditures for infection prevention (≥200 RMB)	1.400	(1.004, 1.951)	0.047*
Past-4-week visiting a doctor	2.813	(1.839, 4.303)	<0.001***
High perceived self-efficacy (Moderately to exactly true)	0.654	(0.460, 0.928)	0.017*
**Ordinal variables**^**c**^	**Adjusted OR**	**95% CI**	***p***
PHQ-15 (0–15)	1.882	(1.409, 2.514)	<0.001***
Meaning in life (1–7)	0.693	(0.520, 0.925)	0.013*
UCLA-3 (3–9)	1.662	(1.220, 2.264)	0.001**
Life satisfaction (1–7)	0.772	(0.544, 0.961)	0.025*
**Continuous variables**^**a**^	**Adjusted *β***	**95% CI**	***p***
EQ5D	-0.090	(-0.112, -0.068)	<0.001***
EQ-VAS	-5.532	(-8.506, -2.559)	<0.001***

EQ5D: European quality of life 5-dimension (EQ5D) Questionnaire; EQ-VAS: European quality of life visual analog scale; Positive GAD-2: anxiety symptoms, two-item Generalized Anxiety Disorder Questionnaire (> = 3); Meaning in life: 1 = utterly meaningless and without purpose, 7 = very purposeful and meaningful; Overall self-recorded negative impact: compared with no or positive impact; Perceived risk for infection: compared with perceived low risk; PHQ-2: Positive depressive symptoms, two-item Patient Health Questionnaire (> = 3); PHQ-15: somatic symptoms, 15-item Patient Health Questionnaire (range: 0–15); PTSD: post-traumatic stress disorder; UCLA-3: three-item UCLA Loneliness Scale (range: 3–9).

^a^Results from linear regression.

^b^Results from binary logistic regression.

^c^Results from ordinal logistic regression. Regressions adjusted for age, gender, marriage, education, income, and job.

*P<0.05

**P<0.01

***P<0.001

Furthermore, participants who had social contacts with COVID-19 patients had a significantly different perception about the epidemic; for example, they reported more worries about infection (OR: 1.844, *p* = 0.009), higher perceived risk of being infected (OR: 3.055, *p*<0.001), and longer perceived time for successful epidemic control (OR: 1.488, *p* = 0.081). Also, they had different health behaviors. For example, they paid more to prevent infection (OR: 1.400, *p* = 0.047) and more likely to have visits to a doctor in the past four weeks (OR: 2.813, *p*<0.001). Finally, they reported lower perceived self-efficacy (OR: 0.654, *p* = 0.017) (Tables 2 and 3).

However, there was no difference in self-rated health, level of social support, and lifestyle factors (e.g., exercise levels, diet (except milk or dairy, beans or bean products), and going out distance) between the participants with or without COVID-19 patients in their social circle (S1 Table).

## Discussion

### Main findings

The current study conducted in mainland China suggests that Chinese people who had social contacts with COVID-19 patients are at an increased risk of having adverse physical and mental outcomes. Furthermore, people who had social contacts with COVID-19 patients had more pessimistic perceptions (e.g., thinking the epidemic as less controllable), reduced self-efficacy, and had increased health utilization (e.g., more likely to visit a doctor). These results highlighted the importance of allocating health resources to this high-risk group.

Despite the lack of similar studies [13, 14], our results were coherent to existing literature. For example, having relatives or friends infected by COVID-19 increased the risk of anxiety disorder among college students in China (OR = 3.007) [14]. Similarly, traveling to an outbreak area increased the risk of suffering from PTSD [13]. Our study showed that not only mental health parameters, but all physical symptoms, perception of our participants, and their health behaviors were affected by having social contacts with COVID-19 patient(s).

The stress or “fight-or-flight” response theory may explain the results [28, 29]. Facing the possibility of infecting with COVID-19, negative emotions, and psychological stresses prompt actions to enhance survival (e.g., buying protective gear/medications). However, when these actions could not eliminate the persistent threat (e.g., living next door with a COVID-19 patient), helplessness and desperation could be developed, which adversely affects people's HRQOL, physical and mental health. Likewise, availability heuristics theory may further explain our results–it is observed that when people evaluate a specific concept, make a decision, or solve a problem, immediate examples are easier to be recalled [30]. Therefore, when dealing with COVID-19, people who had social contacts with COVID-19 patients may recall the information they were exposed to recently (i.e., COVID-19 patients nearby and their illness experience) more easily, which leads to anxiety and fear [30].

### Strengths and limitations

The current study was one of the first to comprehensively assess the pandemic's adverse effects on people who had social contacts with COVID-19 patients. The use of validated questionnaires will also allow for easy comparison with other studies. Quality control was utilized to exclude unreliable answers and responses. This study will contribute to understanding the impacts of COVID-19 in the general population and has identified a high-risk group for targeted screening and interventions.

Yet, some limitations of this study should be discussed. First, the current study used snowball sampling strategies and had recruited participants whose demographics might not represent the general population (for example, most of the participants were highly educated and had a bachelor’s degree). However, the strict social distancing policy during COVID-19 had made other sampling methods infeasible. Second, the cross-sectional nature of the current study may limit the conclusion about causal effects in some scenarios. For example, although it is most likely that having social contacts with COVID-19 patients led to elevated anxiety levels, it is also possible that anxious people defined social contact (i.e., “social circle”) more loosely and subjectively felt a close proximity to the disease or COVID-19 patients. Third, the current cross-sectional design prevented the conclusion about the stability of these physical/mental symptoms and their long-term impacts.

### Perspectives

As the participants who had social contacts with COVID-19 patients had poorer physical and mental health outcomes, proactive strategies to screen and treat diseases should target this high-risk population. During the COVID-19 outbreaks, the delivery of healthcare services was hindered. New delivery methods are being developed, e.g., online interventions and telemedicine, although these are not targeted to people in social contacts with COVID-19 patients [31, 32]. There is currently also a lack of screening strategies for various mental, physical, and social health problems among people who have social contacts with COVID-19 patients in China.

Future studies are suggested to develop strategies to screen for/treat physical and mental illnesses in this high-risk group. Future studies of longer duration (e.g., a cohort study) are also suggested to detect the long-term effect on physical and psychological health. It is currently not clear whether these adverse effects will be resolved after the patients recovered or are cured.

In summary, the current study found that people with confirmed or suspected COVID-19 patients in their social circle were at risk of having adverse health outcomes, including more physical, mental disease symptoms, and a lower HRQOL. Studies with longer duration are needed to understand the long-term impacts. Similarly, strategies to improve health outcomes for these people are needed.

## Supporting information

S1 Table Univariate analysis on self-rated health, social support, lifestyle, and their association with suspected or confirmed infection cases within one's social contacts. Social support: When you need help, can you count on someone willing and able to meet your needs. Chi-square test (χ2) and two-sample t-test were used for categorical data and continuous data respectively. *P<0.05.
(DOCX)

S1 File Original Chinese and translated English questionnaires used in this study. (DOCX)

S2 File Data used in this study. (SAV)
