# Supplementary material for: Physical and mental health outcomes including behavior and attitudes in people having social contacts with COVID-19 patients
Source: PLoS One. 2021 Feb 2;16(2):e0245945. doi: 10.1371/journal.pone.0245945 (PMC7853483; doi:10.1371/journal.pone.0245945)
Supplement: S1 Table — Social support: When you need help, can you count on someone willing and able to meet your needs. Chi-square test (χ2) and two-sample t-test were used for categorical data and continuous data respectively. *P<0.05. (DOCX) [file pone.0245945.s001.docx]

**S1 Table. Univariate analysis on self-rated health, social support, lifestyle, and their association with suspected or confirmed infection cases within one's social contacts**

| **Characteristics** | **Mean±SD or No. (%, 95% CI)** | | | ***p*** |
| --- | --- | --- | --- | --- |
|  | **Total (n=1447)** | **No case within social contacts**  **(n=1274)** | **Cases within social contacts**  **(n=173)** |  |
| Self-rated health (Good to excellent) | 1121 (77.5, 75.2-79.5) | 994 (78.0, 75.7-80.2) | 127 (73.4, 66.4-79.4) | 0.173 |
| Social support (Sometimes to always) | 1381 (95.4, 94.2-96.4) | 1216 (95.4, 94.2-96.5) | 165 (95.4, 91.1-97.6) | 0.966 |
| Vigorous exercise (min/week) | 40.8±76.0 | 40.5±74.7 | 43.2±85.2 | 0.688 |
| Moderate physical exercise (min/week) | 68.0±105.8 | 68.6±106.8 | 63.8±98.4 | 0.555 |
| Sedentary time (h/d) | 7.6±4.0 | 7.6±3.9 | 7.2±4.1 | 0.205 |
| Screen time(h/d) | 6.0±3.0 | 5.9±3.0 | 6.2±3.3 | 0.382 |
| Breakfast (d/week) | 5.5±2.3 | 5.5±2.3 | 5.3±2.4 | 0.495 |
| Fruit (d/week) | 5.2±2.0 | 5.2±2.1 | 5.3±2.0 | 0.895 |
| Vegetables (d/week) | 6.3±1.4 | 6.3±1.4 | 6.2±1.5 | 0.387 |
| Milk or dairy (d/week) | 3.8±2.5 | 3.8±2.5 | 4.4±2.4 | 0.017* |
| Beans or bean products (d/week) | 3.5±2.2 | 3.5±2.2 | 3.9±2.2 | 0.034* |
| Pepper (d/week) | 4.0±2.5 | 4.0±2.6 | 4.0±2.4 | 0.654 |
| Going-out frequency (≥2 times a week) | 753 (52.0, 49.5-54.6) | 667 (52.4, 49.6-55.1) | 86 (49.7, 42.3-57.1) | 0.514 |
| Going-out distance (≥1 kilometer) | 730 (50.4, 47.9-53.0) | 656 (51.5, 48.7-54.2) | 74 (42.8, 35.6-50.2) | 0.031* |

Social support: When you need help, can you count on someone willing and able to meet your needs. Chi-square test (χ2) and two-sample t-test were used for categorical data and continuous data respectively.

**P*<0.05
