# Supplementary material for: Physical and mental health outcomes including behavior and attitudes in people having social contacts with COVID-19 patients
Source: PLoS One. 2021 Feb 2;16(2):e0245945. doi: 10.1371/journal.pone.0245945 (PMC7853483; doi:10.1371/journal.pone.0245945)
Supplement: S1 File — (DOCX) [file pone.0245945.s002.docx]

**中国居民新型冠状病毒传播期间生活质量和身心健康状况调查**

Survey of quality of life, physical and mental health among adults in China during COVID-19

1. {q1_gender}您的性别? Gender

| ○1=男Male |
| --- |
| ○2=女Female |

2. {q2_age}您的年龄（周岁）? Age

_________________________________

3. {q3_marriage}您的婚姻? Marriage

| ○1=已婚Married |
| --- |
| ○2=未婚Singe |
| ○3=再婚Re-married |
| ○4=同居Cohabited |
| ○5=分居Separated |
| ○6=离婚Divorced |
| ○7=丧偶Widowed |

4. {q4_job}您目前的职业为? Job

| ○1=医务人员Medical staff |
| --- |
| ○2=医学生Medical student |
| ○3=农民/渔民Farmer/fisherman |
| ○4=工人/服务人员Worker/service staff |
| ○5=除医务人员以外的专业技术人员/行政管理人员Professional technical personnel/administrative management personnel other than medical personnel |
| ○6=个体户/临时工Self-employed/temporary worker |
| ○7=离退休/病休Retired |
| ○8=无业/失业Unemployed |
| ○9=除医学生以外的其他学生Students other than medical students |
| ○10=家务Housework |
| ○11=其他Others |

5. {q5_edu}您的文化程度是? Education

| ○1=小学及以下Primary school and below |
| --- |
| ○2=初中 Middle school |
| ○3=高中High school |
| ○4=大专College |
| ○5=本科Undergraduate |
| ○6=研究生及以上Postgraduate and above |

6. {q6}您目前人在哪里? Location

| ○1=湖北省武汉市 Wuhan, Hubei |
| --- |
| ○2=湖北省内、武汉市外的其他地区 Other cities in Hubei |
| ○3=湖北省以外的其他省市Other provinces |
| ○4=国外或海外 Overseas |

7.[矩阵单选题] *

|  | 农村Rural | 城市Urban |
| --- | --- | --- |
| 1) 您目前居住在  Current residence{q7_1}： | ○=1 | ○=2 |
| 2) 您过去一年大部分时候居住在residence in the past year{q7_2}： | ○=1 | ○=2 |

8. {q8}您是否育有未成年子女（小于18岁）（含非亲生）? Number of children younger than 18 years old

| ○1=否No |
| --- |
| ○2=是，1名 Yes, one. |
| ○3=是，2名 Yes, two |
| ○4=是，3名或以上 Yes, three or more |

9. {q9}您最近一年的家庭收入，在您平时生活的地方属于什么水平? What is the level of your family income in the last year where you usually live?

| ○1=最高Highest |
| --- |
| ○2=比较高Relatively high |
| ○3=偏高High |
| ○4=中等Average |
| ○5=偏低Low |
| ○6=比较低Relatively low |
| ○7=最低 Lowest |

10. 您此时与谁同住?  Who you live with?

| □{q10_1}配偶Spouse |
| --- |
| □{q10_2}子女 Children |
| □{q10_3}父母 Parents |
| □{q10_4}祖父母 Grandparents |
| □{q10_5}孙子女、外孙Grandchildren |
| □{q10_6}其他人Others |
| □{q10_7}酒店、集体住所Hotels, collective housing |
| □{q10_8}独居Live alone |

12. {q12}您的家人或近亲是否有人感染了新型冠状病毒? Is anyone in your family or close relatives infected with COVID-19?

| ○1=有人确诊感染Yes |
| --- |
| ○2=有人疑似感染，但未确诊Suspected infection but not diagnosed |
| ○3=无人感染No |

13. {q13}您的同事或朋友是否有人感染了新型冠状病毒? Is anyone of your colleagues or friends infected with COVID-19?

| ○1=有人确诊感染Yes |
| --- |
| ○2=有人疑似感染，但未确诊Suspected infection but not diagnosed |
| ○3=无人感染或不知道No |

14. {q14}您所居住的小区/村寨是否有人感染了新型冠状病毒? Is anyone in your community infected with COVID-19?

| ○1=有人确诊感染Yes |
| --- |
| ○2=有人疑似感染，但未确诊Suspected infection but not diagnosed |
| ○3=无人感染或不知道No |

16. {q16}您是否担心自己或家人会被新型冠状病毒感染? Are you worried that you or your family will be infected by COVID-19?

| ○1=已确诊感染Infected |
| --- |
| ○2=非常担心Very worried |
| ○3=担心Worried |
| ○4=不担心Not worried |

17. {q17}您觉得自己感染新型冠状病毒的可能性有多高? How likely do you think you will be infected by COVID-19?

| ○1=已确诊感染Infected |
| --- |
| ○2=非常高Very high |
| ○3=高High |
| ○4=低 Low |
| ○5=非常低 Very low |

18. 过去四周，您有没有出现以下情况:  Symptoms in the past four weeks

| □{q18_1}发热  Fever | □{q18_2}咳痰  Expectoration | □{q18_3}咽痛  Sore throat |
| --- | --- | --- |
| □{q18_4}肌肉酸痛  Muscle ache | □{q18_5}呼吸困难  Difficulty breathing | □{q18_6}眼结膜充血Conjunctival hyperemia |
| □{q18_7}腹泻  Diarrhea | □{q18_8}寒战  Chills | □{q18_9}鼻塞  Stuffy nose |
| □{q18_10}头痛  Headache | □{q18_11}关节酸痛  Joint pain | □{q18_12}胸闷  Chest tightness |
| □{q18_13}恶心  Nausea | □{q18_14}腹痛  Stomach ache | □{q18_15}干咳  Dry cough |
| □{q18_16}流涕  Runny nose | □{q18_17}乏力  Fatigue | □{q18_18}气促  Gasp |
| □{q18_19}胸痛  Chest pain | □{q18_20}呕吐  Vomiting | □{q18_21}头晕  Dizziness |
| □{q18_22}无以上任何症状None |  |  |

19.过去四周，您是否受以下情况困扰? Over the last week, how often have you been bothered by the following?

1) {q19_1}胃/肚痛 Stomach pain

| ○1=没有困扰Not at all |
| --- |
| ○2=少许困扰Bothered a little |
| ○3=很多困扰Bothered a lot |

2) {q19_2}背痛 Back pain

| ○1=没有困扰Not at all |
| --- |
| ○2=少许困扰Bothered a little |
| ○3=很多困扰Bothered a lot |

3) {q19_3}胳膊、 腿或关节疼痛（膝、髋关节等）Pain in arms, legs or joints (knees, hips, etc.)

| ○1=没有困扰Not at all |
| --- |
| ○2=少许困扰Bothered a little |
| ○3=很多困扰Bothered a lot |

4) {q19_4}痛经或其他月经问题 Menstrual cramps or other problems you’re your periods

| ○1=没有困扰Not at all |
| --- |
| ○2=少许困扰Bothered a little |
| ○3=很多困扰Bothered a lot |
| ○-3=跳过Skip |

5) {q19_5}性生活中有疼痛或其他问题Pain or problems during sexual intercourse

| ○1=没有困扰Not at all |
| --- |
| ○2=少许困扰Bothered a little |
| ○3=很多困扰Bothered a lot |

6) {q19_6}头痛Headache

| ○1=没有困扰Not at all |
| --- |
| ○2=少许困扰Bothered a little |
| ○3=很多困扰Bothered a lot |

7) {q19_7}胸痛Chest pain

| ○1=没有困扰Not at all |
| --- |
| ○2=少许困扰Bothered a little |
| ○3=很多困扰Bothered a lot |

8) {q19_8}头晕Dizziness

| ○1=没有困扰Not at all |
| --- |
| ○2=少许困扰Bothered a little |
| ○3=很多困扰Bothered a lot |

9) {q19_9}短时间昏倒Fainting spells

| ○1=没有困扰Not at all |
| --- |
| ○2=少许困扰Bothered a little |
| ○3=很多困扰Bothered a lot |

10) {q19_10}感觉心跳加重或急速地跳动Feeling your heart pound or race

| ○1=没有困扰Not at all |
| --- |
| ○2=少许困扰Bothered a little |
| ○3=很多困扰Bothered a lot |

11) {q19_11}呼吸急促Shortness of breath

| ○1=没有困扰Not at all |
| --- |
| ○2=少许困扰Bothered a little |
| ○3=很多困扰Bothered a lot |

12) {q19_12}便秘、肠道不适、或腹泻Constipation, loose bowels or diarrhea

| ○1=没有困扰Not at all |
| --- |
| ○2=少许困扰Bothered a little |
| ○3=很多困扰Bothered a lot |

13) {q19_13}恶心、胀气、或消化不良nausea, has or indigestion

| ○1=没有困扰Not at all |
| --- |
| ○2=少许困扰Bothered a little |
| ○3=很多困扰Bothered a lot |

14) {q19_14}感到疲劳或精力不足Feeling tired or having low energy

| ○1=没有困扰Not at all |
| --- |
| ○2=少许困扰Bothered a little |
| ○3=很多困扰Bothered a lot |

15) {q19_15}睡眠有问题Trouble sleeping

| ○1=没有困扰Not at all |
| --- |
| ○2=少许困扰Bothered a little |
| ○3=很多困扰Bothered a lot |

20. {q20}您过去四周内有没有就诊过? Have you seen a doctor in the past four weeks?

| ○1=有，网上就诊Yes, online |
| --- |
| ○2=有，诊所、医院就诊Yes, in clinic or hospital |
| ○3=无 No |

21.下面列举了一些人们可能会有的情况，请选择适合您的答案Here are some situations that people may have, please choose the answer that suits you
1) {q21_1}近两周我做事提不起劲或没有兴趣 Little interest or pleasure in doing things in the past two weeks?

| ○1=没有Not at all |
| --- |
| ○2=有几天Several days |
| ○3=一半以上时间More than half the days |
| ○4=几乎每天Nearly every day |

2) {q21_2}近两周我感到情绪低落、沮丧或绝望 Feeling down, depressed, or hopeless in the past two weeks

| ○1=没有Not at all |
| --- |
| ○2=有几天Several days |
| ○3=一半以上时间More than half the days |
| ○4=几乎每天Nearly every day |

3) {q21_3}近两周有不如死掉或用某种方式伤害自己的念头 Thoughts that you would be better off dead, or of hurting yourself in some way in the past two weeks

| ○1=没有Not at all |
| --- |
| ○2=有几天Several days |
| ○3=一半以上时间More than half the days |
| ○4=几乎每天Nearly every day |

22. {q22}近两周我感到不安、担心或烦躁 Feeling nervous, anxious, or on edge in the past two weeks

| ○1=没有Not at all |
| --- |
| ○2=有几天Several days |
| ○3=一半以上时间More than half the days |
| ○4=几乎每天Nearly every day |

23. {q23}近两周我不能停止担心或控制不了担心 Not being able to stop or control worrying in the past two weeks

| ○1=没有Not at all |
| --- |
| ○2=有几天Several days |
| ○3=一半以上时间More than half the days |
| ○4=几乎每天Nearly every day |

24. {q24}您有没有反复不安地、控制不住地想到或梦到新冠肺炎有关的事件? Have you repeatedly thought about or dreamed about events related to COVID-19?

| ○1=没有Absent |
| --- |
| ○2=轻度Mild / subthreshold |
| ○3=中度Moderate / threshold |
| ○4=重度Severe / markedly elevated |
| ○5=极重Extreme / incapacitating |

25. {q25}您有没有回避与新冠肺炎有关的信息、人、活动、地点、想法、感觉等 Have you avoided information, people, activities, places, thoughts, and feelings related to COVID-19?

| ○1=没有Absent |
| --- |
| ○2=轻度Mild / subthreshold |
| ○3=中度Moderate / threshold |
| ○4=重度Severe / markedly elevated |
| ○5=极重Extreme / incapacitating |

26. {q26}您是否觉得自己缺乏人陪伴? How often do you feel that you lack companionship?

| ○1=几乎没有Rarely |
| --- |
| ○2=有时Sometimes |
| ○3=经常Often |

27. {q27}您是否觉得被忽略? How often do you feel left out?

| ○1=几乎没有Rarely |
| --- |
| ○2=有时Sometimes |
| ○3=经常Often |

28. {q28}您是否觉得自己被其他人孤立? How often do you feel isolated from others?

| ○1=几乎没有Rarely |
| --- |
| ○2=有时Sometimes |
| ○3=经常Often |

29. {q29}当您需要帮助时，有没有一位愿意及能够满足您需求的人? When you need help, is there someone who is willing and able to meet your needs?

| ○1=总是Often |
| --- |
| ○2=有时Sometimes |
| ○3=从来没有Never |

30. {q30}您觉得自己个人的存在是What’s your meaning of personal existence

1分表示“完全没有意义和目的utterly meaningless and without purpose”，7分表示“十分有意义和目的very purposeful and meaningful”

| ○1 | ○2 | ○3 | ○4 | ○5 | ○6 | ○7 |
| --- | --- | --- | --- | --- | --- | --- |

31. {q31}无论什么事在您身上发生，您都能够应付自如。I can usually handle whatever comes my way

| ○1=完全不正确Not at all true |
| --- |
| ○2=小部分正确 Partly true |
| ○3=多数正确 Mostly true |
| ○4=完全正确Exactly true |

32. {q32}您是否满意自己的生活? Are you satisfied with your life?

| ○1=非常不满意Very dissatisfied |
| --- |
| ○2=不满意 Dissatisfied |
| ○3=有一点不满意A little dissatisfied |
| ○4=中立Average |
| ○5=有一点满意A little satisfied |
| ○6=满意Satisfied |
| ○7=非常满意Very satisfied |

36. {q36}行动能力（指您如果被允许的前提下的走动能力） MOBILITY

| ○1=我可以四处走动，没有任何问题。I have no problems in walking about |
| --- |
| ○2=我的行动有轻微问题。I have slight problems in walking about |
| ○3=我的行动有中度问题。I have moderate problems in walking about |
| ○4=我的行动有严重问题。I have severe problems in walking about |
| ○5=我无法行动。I am unable to walk about |

37. {q37}自我照顾 SELF-CARE

| ○1=我在洗澡或穿衣方面没有任何问题。I have no problems washing or dressing myself |
| --- |
| ○2=我在洗澡或穿衣方面有轻微问题I have slight problems washing or dressing myself |
| ○3=我在洗澡或穿衣方面有中度问题。I have moderate problems washing or dressing myself |
| ○4=我在洗澡或穿衣方面有严重问题。I have severe problems washing or dressing myself |
| ○5=我无法自己洗澡或穿衣。I am unable to wash or dress myself |

38. {q38}平常活动(如工作、读书、家务、家庭或休闲活动) USUAL ACTIVITIES (e.g. work, study, housework, family or leisure activities)

| ○1=我能进行平常活动，没有任何问题。I have no problems doing my usual activities |
| --- |
| ○2=我在进行平常活动方面有轻微问题。I have slight problems doing my usual activities |
| ○3=我在进行平常活动方面有中度问题。I have moderate problems doing my usual activities |
| ○4=我在进行平常活动方面有严重问题。I have severe problems doing my usual activities |
| ○5=我无法进行平常活动。I am unable to do my usual activities |

39. {q39}疼痛 / 不舒服 PAIN / DISCOMFORT

| ○1=我没有任何疼痛或不舒服。I have no pain or discomfort |
| --- |
| ○2=我觉得轻微疼痛或不舒服。I have slight pain or discomfort |
| ○3=我觉得中度疼痛或不舒服。I have moderate pain or discomfort |
| ○4=我觉得严重疼痛或不舒服。I have severe pain or discomfort |
| ○5=我觉得极度疼痛或不舒服。I have exterme pain or discomfort |

40. {q40}焦虑 / 沮丧 ANXIETY/DEPRESSION

| ○1=我不觉得焦虑或沮丧。I am not anxious or depressed |
| --- |
| ○2=我觉得轻微焦虑或沮丧。I am slightly anxious or depressed |
| ○3=我觉得中度焦虑或沮丧。I am moderately anxious or depressed |
| ○4=我觉得严重焦虑或沮丧。I am severely anxious or depressed |
| ○5=我觉得极度焦虑或沮丧。I am extremely anxious or depressed |

41.我们想知道您今天的健康状况有多好或多坏。We would like to know how good or bad your health is TODAY.

0表示您能想象到的最坏的健康状况。
100表示您能想象到的最好的健康状况。

100 means the best health you can imagine.

0 means the worst health you can imagine.

请打一个分数，以显示您今天的健康状况如何 Please give a score to show how well your health is today

{q41}_________________________________

42. 在过去七日，您做剧烈运动的时间总共有多长呢?（剧烈运动是指做完后，呼吸会非常急速，例如跑步、跳操、踢足球、游泳、做粗重工作等。）In the past seven days, how long did you spend doing vigorous exercise in total? (Vigorous exercise refers to the rapid breathing after you finish, such as running, aerobics, playing football, swimming, doing heavy work, etc.){q42}_________分钟 minutes

43. 在过去七日，您做中等强度的体能活动的时间总共有多长呢?（中等强度体能活动是指做完后，呼吸会比平常急速，例如踏单车、洗车打腊、快步走、擦窗等。）In the past seven days, how long did you spend doing moderate-intensity physical activities in total? (Moderate-intensity physical activity means that your breathing will be faster than usual after you do it, such as cycling, car washing and waxing, brisk walking, rubbing Windows etc.){q43}__________分钟 minutes

44. 在过去七日，清醒的时候，您有多少时间是坐着或者躺着的呢? In the past seven days, when awake, how much time did you sit or lie down?
平均{q44}________小时/天  Average __________hours/day

49. 您最近一星期有多少天吃以下食物? How many days did you eat the following foods in the last week?

|  | 0天 | 1天 | 2天 | 3天 | 4天 | 5天 | 6天 | 7天 |
| --- | --- | --- | --- | --- | --- | --- | --- | --- |
| 1) 早餐Breakfast{q49_1} | ○=0 | ○=1 | ○=2 | ○=3 | ○=4 | ○=5 | ○=6 | ○=7 |
| 2) 水果Fruits{q49_2} | ○=0 | ○=1 | ○=2 | ○=3 | ○=4 | ○=5 | ○=6 | ○=7 |
| 3) 蔬菜Vegetables{q49_3} | ○=0 | ○=1 | ○=2 | ○=3 | ○=4 | ○=5 | ○=6 | ○=7 |
| 4) 牛奶或奶制品Milk or dairy products {q49_4} | ○=0 | ○=1 | ○=2 | ○=3 | ○=4 | ○=5 | ○=6 | ○=7 |
| 5) 豆类或豆制品Beans or soy products  {q49_5} | ○=0 | ○=1 | ○=2 | ○=3 | ○=4 | ○=5 | ○=6 | ○=7 |
| 6) 辣椒Chili  {q49_6} | ○=0 | ○=1 | ○=2 | ○=3 | ○=4 | ○=5 | ○=6 | ○=7 |

50. {q50}最近2周，您外出的频率是? In the past 2 weeks, how often did you go out?

| ○1=从未出门Never |
| --- |
| ○2=少于一周一次Less than once a week |
| ○3=一周1次Once a week |
| ○4=一周2-3次 Two to three times a week |
| ○5=一周4-5次Four to five times a week |
| ○6=几乎每天 Almost every day |

51. {q51}最近2周，您的活动范围半径? What is the radius of your activity in the last 2 weeks?

| ○1= <100米 Less than 100 meters |
| --- |
| ○2= 100-499米 100-499 meters |
| ○3= 500-999米 500-999 meters |
| ○4= 1000-1999米 1000-1999 meters |
| ○5= 2公里-4.99公里 2-4.99 kilometers |
| ○6= 5公里-9.99公里 5-9.99 kilometers |
| ○7= 10公里-49公里 10-49 kilometers |
| ○8= 50公里及以上 More than 50 kilometers |

52. {q52}最近2周，您平均每日的手机、网络、电视、游戏机的使用时间: In the last 2 weeks, your average daily usage time of mobile phones, internet, TV, and game consoles:

| ○1=无 No |
| --- |
| ○2=少于1小时 Less than an hour |
| ○3=1-2小时 1-2 hours |
| ○4=3-4小时 3-4 hours |
| ○5=5-6小时 5-6 hours |
| ○6=7-8小时7-8 hours |
| ○7= 9-10小时 9-10 hours |
| ○8=10小时以上 More than 10 hours |

56. {q56}自2019年12月以来，您个人用于预防或治疗新冠肺炎的费用为（包括口罩、消毒液、药物等）: Since December 2019, what is your personal expenses for the prevention or treatment of COVID-19 (including masks, disinfectants, drugs, etc.)

| ○1=无 None |
| --- |
| ○2=1-199元(¥ yuan) |
| ○3=200-499元(¥ yuan) |
| ○4=500-999元(¥ yuan) |
| ○5=1000-1499元(¥ yuan) |
| ○6=1500-1999元(¥ yuan) |
| ○7=2000元或以上 More than 2000 yuan |

57. {q57}自2019年12月以来，您是否因为新冠肺炎疫情而耽误其他健康问题的诊治（如心脏病，糖尿病，肿瘤等）? Since December 2019, have you delayed the diagnosis and treatment of other health problems (such as heart disease, diabetes, tumors, etc.) due to the COVID-19 epidemic?

| ○1=没有需要诊疗的健康问题No health problems requiring medical treatment |
| --- |
| ○2=有，但得到了及时诊治（包括线上和线下）Yes, and received timely diagnosis and treatment (including online and offline) |
| ○3=有，已经延期 Yes, delayed |
| ○4=有，不能延期且诊疗受阻Yes, it cannot be delayed and cannot be treated |

14) {q58_14}整体而言，目前新冠肺炎疫情对您各方面的影响程度：Overall, the current degree of impact of the COVID-19 epidemic on you in all aspects:

| ○1=负面影响，非常大 Very negative impact |
| --- |
| ○2=负面影响，比较大Relatively large negative impact |
| ○3=负面影响，比较小Relatively small negative impact |
| ○4=无影响 No impact |
| ○5=正面影响，比较小Relatively small posotive impact |
| ○6=正面影响，比较大Relatively large positive impact |
| ○7=正面影响，非常大Very positive impact |

60. {q60}您估计要多少时间国家新型冠状病毒的疫情就可以被完全控制住? How long do you estimate that the country's COVID-19 outbreak can be completely controlled?

| ○1= 1-2个月 1-2 months |
| --- |
| ○2= 3-6个月 3-6 months |
| ○3= 半年-1年 Half to one year |
| ○4= 1-2年 1-2 years |
| ○5= 3年以上 More than 3 years |

62. {q62}总括来说，您认为您的健康状况是: In general, how would you rate your health

| ○1=极好Very good |
| --- |
| ○2=很好 Good |
| ○3=好 Moderate |
| ○4=一般 Bad |
| ○5=差 Very bad |
